# Spatiotemporal control of cell–cell reversible interactions using molecular engineering

**DOI:** 10.1038/ncomms13088

**Published:** 2016-10-06

**Authors:** Peng Shi, Enguo Ju, Zhengqing Yan, Nan Gao, Jiasi Wang, Jianwen Hou, Yan Zhang, Jinsong Ren, Xiaogang Qu

**Affiliations:** 1 Laboratory of Chemical Biology and State Key Laboratory of Rare Earth Resource Utilization, Changchun Institute of Applied Chemistry, Chinese Academy of Sciences, Changchun 130022, China; 2 University of Chinese Academy of Sciences, Beijing 100039, China; 3 College of life science, Jilin University, Changchun 130012, China

## Abstract

Manipulation of cell–cell interactions has potential applications in basic research and cell-based therapy. Herein, using a combination of metabolic glycan labelling and bio-orthogonal click reaction, we engineer cell membranes with β-cyclodextrin and subsequently manipulate cell behaviours via photo-responsive host-guest recognition. With this methodology, we demonstrate reversible manipulation of cell assembly and disassembly. The method enables light-controllable reversible assembly of cell–cell adhesion, in contrast with previously reported irreversible effects, in which altered structure could not be reused. We also illustrate the utility of the method by designing a cell-based therapy. Peripheral blood mononuclear cells modified with aptamer are effectively redirected towards target cells, resulting in enhanced cell apoptosis. Our approach allows precise control of reversible cell–cell interactions and we expect that it will promote further developments of cell-based therapy.

## Abstract (web summary)

Reversible manipulation of cell-cell interactions has potential applications in basic research and cell-based therapy. Here the authors control cell-cell adhesion *in vitro* with light, by modifying the surface sugars of cells to display β-cyclodextrin, which recognises one isoform of light-isomerizable azobenzene linkers.

## Graphical abstract

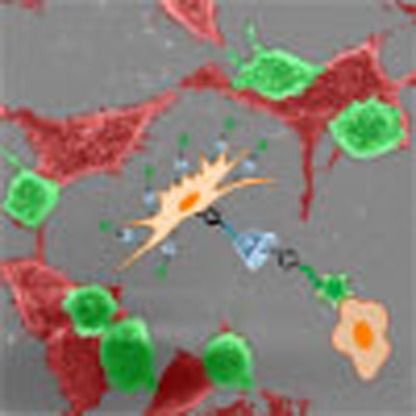

Dynamic cell–cell interactions are imperative for correct cell behaviour. The failure of cell communications can cause uncontrollable cell growth and cancer^1^,^2^. Responding cells make direct physical contact with signalling cells, ‘read' their markers and give appropriate responses. For example, intercellular proximity is a critical step towards antigen presentation. Immune cells detect antigen presented on infected cell surfaces, triggering cytokine release, causing lysis or apoptosis. Therefore, spatiotemporal modulation of cell–cell interactions would benefit fundamental cell-behavioural studies, and allow unprecedented control of cell behaviour, as well as provide synthetic biological method for the design of cell-based therapy^3^,^4^,^5^.

Apart from molecular biological techniques to genetically engineer cells^6^,^7^, in recent years, a number of non-genetic cell-surface engineering methods have been devised for the control of ligand presentation on cell surfaces^8^, which would greatly facilitate the ability to manipulate cellular interactions. Among them, biotin–streptavidin bridge is a general strategy, where the surfaces of two cell types are modified with a biotin–streptavidin pair, followed by the assembly of the modified cells via specific biotin–streptavidin interactions^9^,^10^. DNA has also been used as a bonding agent for cell–cell contacts^11^,^12^. By taking advantage of metabolic labelling approach to modify cell surfaces with complementary short oligonucleotides, DNA hybridization assay has been reported to control over cell–cell interactions^11^. Besides, lipid–DNA aptamer conjugates have been used to modulate cell–cell adhesion on receptor–ligand binding^12^. Recently, methodology of liposome-to-cell fusion has been developed for delivery of bioorthogonal chemical groups to tailor cell membranes and subsequently direct the formation of multilayered cell tissues^13^,^14^,^15^. Lipid chemically self-assembled nanorings could be designed as a molecular scaffold to engineer cell surfaces and temporally control cell–cell interactions^16^. Thus far, the cell surfaces have been engineered to respond to temperature^11^, enzymolysis^12^, redox potential^14^ and chemical stimuli^16^, which can be used for modulating intercellular contacts. Although promising, it is still a challenge to control cell-cell interactions in time and space. Light manipulation may provide solution to this issue as it allows control over the cells from a distance with relatively high spatial and temporal precision^17^,^18^. However, the existing method relies on irreversible control, that is, once the designed structure on cell surface is altered, it cannot be regenerated for further use^15^. This can be overcome by engineering a photo-switchable cell surface.

Azobenzene represents a well-known class of photo-switchable compounds, the two isomers of which, the *trans* and *cis* forms, can be reversibly interconverted on photoirradiation^19^. Also, the molecular recognition of azobenzenes with cyclodextrins (CD) could be reversibly controlled by photoirradiation: the rodlike *trans* isomer forms a stable inclusion complex with CD, while the bent *cis* isomer does not fit in CD^20^,^21^. The reversible photoisomerization of azobenzene has been used for dynamic control of cells and bacteria capture/release on stimuli-responsive substrates^22^,^23^. Herein, for the first time, we extended this highly efficient photo-driven supramolecular recognition for spatio-temporal manipulation of cell-cell reversible interactions.

To realize this, tailoring cell surfaces with β-CD is a prerequisite. Non-covalent cell-surface modification approaches based on lipid insertion and liposome-to-cell fusion have received increasing attention^4^,^5^,^12^,^13^,^14^,^15^,^16^. Although such methods are simple and efficient, using lipid anchor may suffer from the stability problem due to the dynamic nature of the phospholipid membrane. Metabolic labelling approaches have been well employed to introduce different functional groups on cell surfaces, showing powerful applications in cell surface engineering^24^,^25^. Unnatural monosaccharide derivatives are metabolically incorporated into cell-surface glycans, resulting in the cell surface display of bioorthogonal groups as specific chemical handles. Therefore, a series of functional components such as probes^26^,^27^,^28^,^29^, biomolecules^30^, and nanomaterials^31^,^32^, can be covalently attached via bioorthogonal reactions.

Herein, we take advantage of metabolic labelling approach and bio-orthogonal click reaction to tailor cell membranes with host molecules (Fig. 1a). The strategy involve feeding cells peracetylated N-azidoacetylgalactosamine (Ac_4_GalNAz) to enrich cell surface glycoconjugates with the azide tag^33^, followed by conjugating with alkynyl and PEG-modified β-CD (alkynyl-PEG-β-CD) via a bio-orthogonal copper(I)-catalysed azide-alkyne cycloaddition (CuAAC). The β-CD modifications enable dynamic control of ligand presentation on cell membranes. If azobenzene is incorporated as switchable recognition component, as indicated in Fig. 1b, we could construct a photo-controlled reversible system. With this methodology, we firstly investigate reversible manipulation of cell assembly and disassembly with a homobifunctional cross-linking agent. Furthermore, considering that aptamers are promising recognition elements with high binding affinity to a broad range of targets, including cells^34^,^35^, we reason azobenzene-labelled aptamers (azo-aptamer) anchored on the cell surface could act as targeting ligands and induce cell–cell adhesion. It should be noted that this photo-active cell surface engineering method allows modulating intercellular contacts in space and time. More importantly, applying photo-driven host–guest recognition affords the cell–cell adhesion a novel property of reversible assembly controlled by light^12^, which is distinct from the irreversible effects, such as photolytic damage of original structure^15^. At last, we illustrate an actual use by designing a cell-based therapy. Peripheral blood mononuclear cells (PBMCs) modified with aptamer are effectively redirected towards target cells, resulting in enhanced cell apoptosis. Our approach would allow unprecedented control of cell behaviour and benefit studies of contact-dependent cell-cell communications.

## Results

### Cell surfaces modification with β-CD

**Figure 1:**
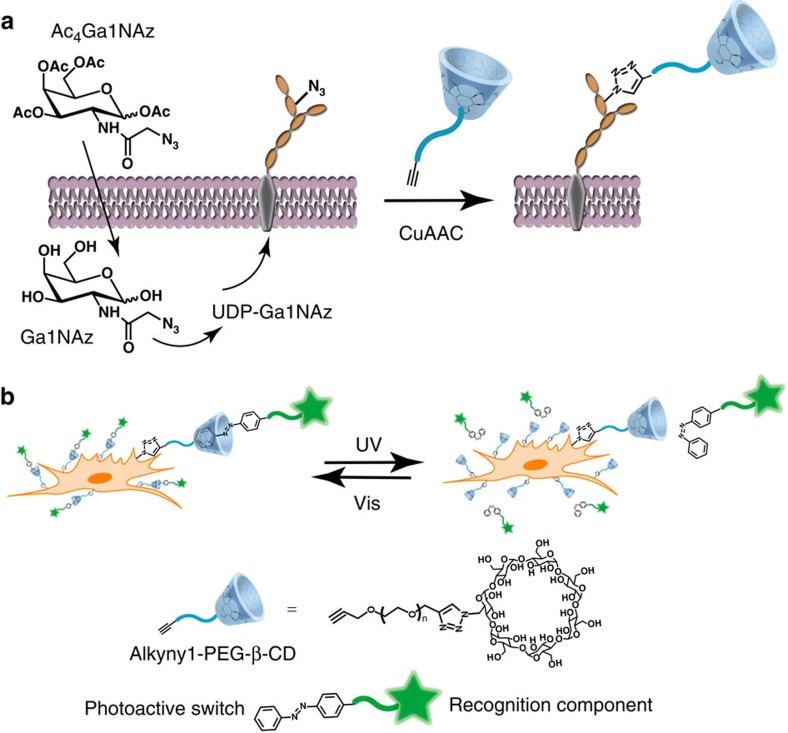
Schematic illustration of engineering photo-responsive host-guest recognition on cell surfaces. (**a**) Metabolic labelling of mucin-type O-glycans with Ac_4_GalNAz by the GalNAc salvage pathway resulted in the enrichment of the azide tag. CuAAC was used to conjugate alkynyl-PEG-β-CD. (**b**) Recognition component-linked azobenzenes could be built onto β-CD-conjugated cell surface through host–guest interactions to construct photo-controlled reversible systems.

In our strategy, the metabolic substrate Ac_4_GalNAz was selected on the basis of its known incorporation into mucin-type O-linked glyco-proteins in mammalian cells via the N-acetylgalactosamine (GalNAc) salvage pathway (Fig. 1a)^33^. As GalNAc was also found in certain glycolipids, chondroitin sulfate proteoglycans and as a terminal modification of some N-linked glycans, these structures might also be labelled with Ac_4_GalNAz^36^. MCF-7 human breast cancer cells were treated with Ac_4_GalNAz in culture media for three days, and the presence of azide groups on the cell surface was determined by covalent attachment of an alkynyl-based probe, FAM alkyne (Supplementary Fig. 1a). As shown in confocal fluorescence images, bright fluorescence was observed for the Ac_4_GalNAz-treated cells when tagged with FAM alkyne by the click reaction (Supplementary Fig. 1b). In contrast, no fluorescence signal was observed for cells not exposed to Ac_4_GalNAz (Supplementary Fig. 1c). These results confirmed Ac_4_GalNAz-treated cells successfully expressed cell surface–associated azide groups and could be chemoselectively decorated with alkynyl conjugates.

**Figure 2:**
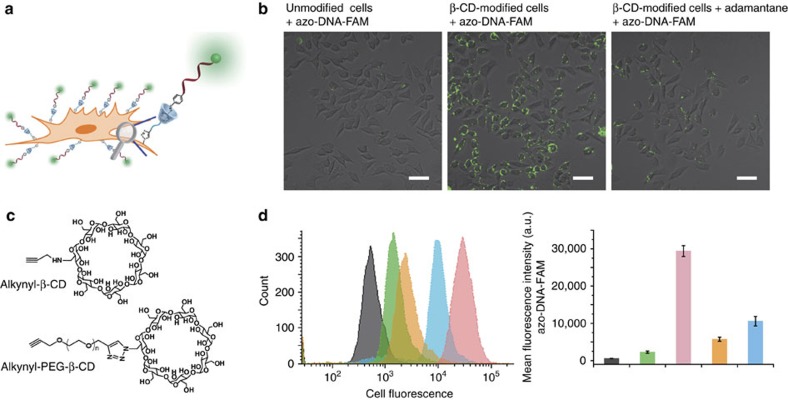
Characterization of cell-surface modification by CLSM images and flow cytometry analysis. (**a**) Schematic illustration of cell-surface decoration with azo-DNA-FAM. (**b**) CLSM images of unmodified or β-CD-modified MCF-7 cells after different treatment. FAM signals were found only on the cell surface. Scale bars, 50 μm. (**c**) Chemical structure of alkynyl-β-CD and alkynyl-PEG-β-CD. (**d**) Flow cytometry assay of azo-DNA-FAM binding efficiency, black: untreated MCF-7 cells, green: unmodified cells treated with azo-DNA-FAM, pink: alkynyl-PEG-β-CD modified cells treated with azo-DNA-FAM, orange: alkynyl-PEG-β-CD modified cells treated with adamantine followed by azo-DNA-FAM, blue: alkynyl-β-CD modified cells treated with azo-DNA-FAM. Data were presented as mean±s.d (*n*=3).

Next, to tailor cell surface with β-CD, azides installed within cell surface glycoconjugates was subjected to TBTA-assisted CuAAC with alkynyl-PEG-β-CD (Supplementary Fig. 2) to produce stable cell-surface adducts. A series of experiments were performed to demonstrate the successful β-CD modification and the ability of β-CD moiety to include guests. Firstly, we modified azobenzene motif onto a FAM-labelled DNA (azo-DNA-FAM) and hypothesized that azo-DNA-FAM recognized β-CD on the cell surface and lighted up the cell membrane (Fig. 2a). As can be seen from confocal laser scanning microscopy (CLSM) images and flow cytometry analysis (Fig. 2b,d), after incubation with azo-DNA-FAM, obvious fluorescence was observed in β-CD-modified cells. FAM signals were localized on the cell membrane. In contrast, control experiments carried out with unmodified cells showed negligible fluorescence. Moreover, competition experiments, using adamantane, were performed. Due to the higher association constant for β-CD-adamantine complex (10^4^–10^5^ M^−1^), azobenzene binding could be blocked by adamantine. As expected, no distinct azo-DNA-FAM binding was observed for β-CD-modified cell exposed to adamantine. These results confirmed the successful β-CD modification and azobenzene binding. We thereafter quantified the number of cell-bound β-CD through quantification of azo-DNA-FAM. β-CD modified cells were treated with azo-DNA-FAM, and subsequently analysed by flow cytometry. The mean fluorescence intensities of cells were compared with known standards (Quantum FITC MESF, Bangs Laboratories) to determine the number of azo-DNA-FAM per cell^11^. Control experiment of unmodified cells incubated with azo-DNA-FAM was carried out to aid in interpreting fluorescence intensity results. The results showed that ∼1.5 × 10^6^ molecules were modified onto the cell surface (Supplementary Fig. 3). Surface β-CD density was about 4,787 molecules per μm^2^. To further determine the half-life of β-CD on cell surface, we labelled remaining surface-associated β-CD with azo-DNA-FAM over 72 h^5^. The results showed that the surface half-life was about 17.4 h (Supplementary Fig. 4).

According to the previous reports^12^,^16^,^37^, PEG linker was able to reduce nonspecific and steric interactions between the cell-surface molecules and the modified groups, thus enabling the modified molecules to protrude from the cell surface and maintain its functional conformation. To investigate the effect of PEG linker, we prepared alkynyl-β-CD (without PEG spacer) as a control (Supplementary Fig. 5). Ac_4_GalNAz-treated MCF-7 cells were tagged with alkynyl-PEG-β-CD or alkynyl-β-CD by a click reaction, and subsequently coupled to azo-DNA-FAM. The cell surface labelling was explored using flow cytometry analysis. Figure 2d showed that fluorescence signals in alkynyl-β-CD modified cells were much lower than that of alkynyl-PEG-β-CD modified cells, which could be possibly attributed to the lower β-CD modification efficiency or lower azobenzene binding efficiency in alkynyl-β-CD treated cells. In general, the above results indicate that PEG linkers play an important role in cell-surface modification. In all experiments otherwise stated, we used alkynyl-PEG-β-CD.

**Figure 3:**
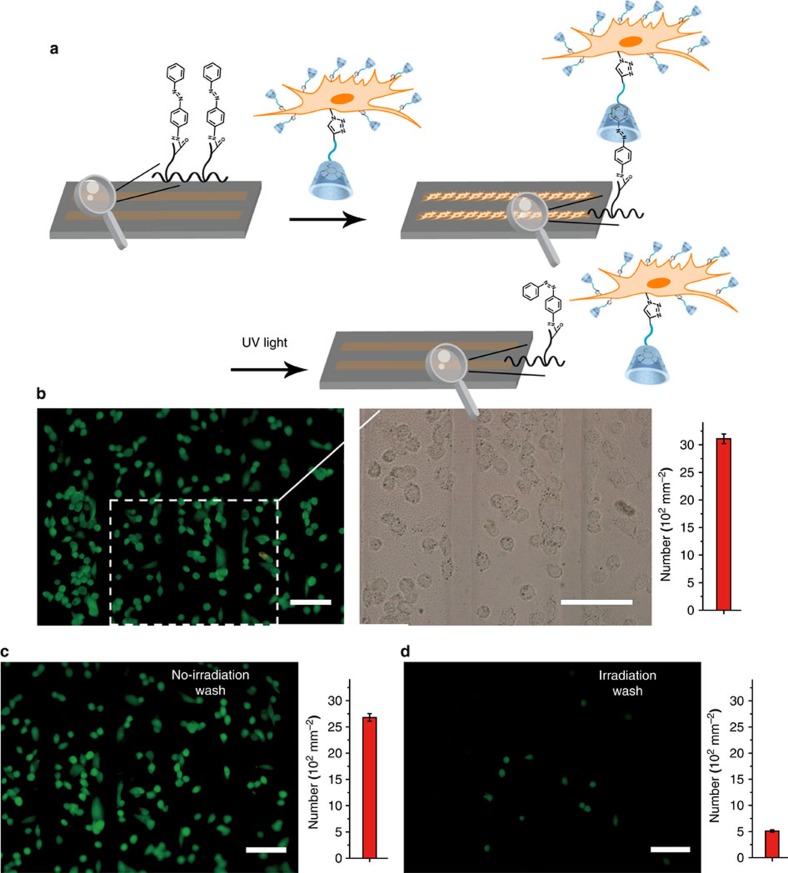
Fluorescence images and quantitative analysis of controlled cell adhesion and release. (**a**) Outline of attaching β-CD-labelled cells to an azobenzene-patterned substrate and releasing them again. (**b**) Fluorescence image (left) and bright-field image at higher magnification (right) indicated β-CD-labelled cells could selectively attach to *trans*-azobenzene-patterned regions. Cells on the substrate were treated with (**d**) or without (**c**) ultraviolet irradiation (365 nm, 15 W, 10 min) for the selective release. Scale bars, 50 μm. Data were presented as mean±s.d. from three independent experiments.

We next investigated the utility of β-CD on the cell surface by attaching cells to an azobenzene-patterned substrate (Supplementary Fig. 6) and releasing them again (Fig. 3a). β-CD-labelled cells were seeded to patterned substrate and cultured for 4 h, thereafter washed with PBS and stained with green dyes. As shown in Fig. 3b, the β-CD-labelled cells could selectively attach to *trans*-azobenzene-patterned regions through host–guest interactions. As a control, unmodified cells did not attach to the surface (Supplementary Fig. 7). Ultraviolet light trigger was then applied to induce the isomerization of azobenzene, from *trans*-isomer to the *cis*-isomer. As a result, cells would detach from the substrate because of the mismatch between the host and guest. As expected, just ∼14% of the adherent cells were released without ultraviolet irradiation. However, after exposure to ultraviolet light and washing with PBS, ∼84% of the original adherent cells were detached from the substrate. (Fig. 3c,d). Such a strategy enables a synthetic approach to spatio-temporally manipulate cells in 2D and may be extended for tissue engineering applications. Besides, we prepared azobenzene and PEG-modified fluorescent silica nanoparticle (azo-PEG-SiNP, Supplementary Fig. 8) and tested the reversible binding of azo-PEG-SiNP to the β-CD-modified cell surfaces (Supplementary Fig. 9a). The β-CD-labelled cells were first fixed, then incubated with azo-PEG-SiNP for 20 min, washed three times by PBS and imaged on confocal fluorescence microscope. The results demonstrated modified cell surfaces were lighted up by azo-PEG-SiNP (Supplementary Fig. 9b). Scanning electron microscopy (SEM) also confirmed attachment of nanoparticles on the cell surfaces (Supplementary Fig. 9c). In contrast, azo-PEG-SiNP showed minimal binding to non-labelled cells under identical conditions as illustrated by negligible fluorescence (Supplementary Fig. 10). Importantly, noncovalent nature of host–guest interactions allowed detachment of the imaging agent. On ultraviolet irradiation, azo-PEG-SiNP was released from the cell surfaces and negligible labelling was observed (Supplementary Fig. 10d). The photocontrolled attachment and detachment of azo-PEG-SiNP also confirmed the successful presentation of β-CD on cell surface.

### Reversible manipulation of cell assembly and disassembly

**Figure 4:**
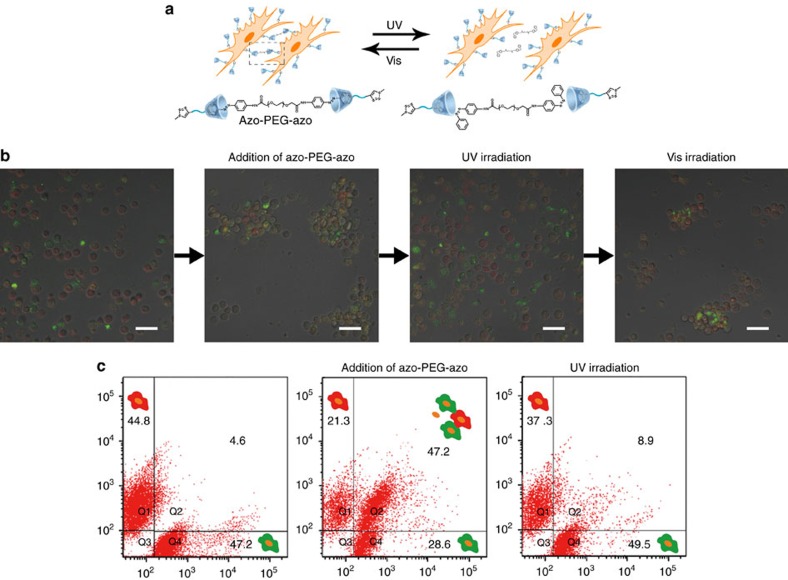
Reversible manipulation of cell assembly and disassembly. (**a**) Schematic illustration describing reversible cell–cell contacts. A homobifunctional guest molecule served as ‘reversible cell glues' could induce assembly and disassembly of β-CD-modified cells through light-manipulation. The rod-like *trans*-isomer formed a stable inclusion complex with β-CD, while the bent *cis*-isomer did not fit in β-CD. (**b**) CLSM images and (**c**) flow cytometry analysis of β-CD-modified cells with different treatment. Scale bars, 50 μm.

The presentation of β-CD on the cell membrane enabled controlled display of functional components through host–guest interactions. We extended this methodology to demonstrate dynamic control over cell–cell interactions. Before the experiments, we checked the cell viability before and after bio-orthogonal click reaction. Ac_4_GalNAz treatment had little effect on cell viability at a nontoxic concentration of 50 × 10^−6^ M. The subsequent modification with alkynyl-PEG-β-CD resulted in a slight decrease of cell viability. Besides, the viability of the cells did not change obviously when treated with 10 min ultraviolet irradiation (365 nm, 15 W) (Supplementary Fig. 11). Next, we used a homobifunctional guest molecule (azo-PEG-azo) which carried two identical supramolecular binding sites: azobenzene groups (Supplementary Fig. 12). By means of isothermal titration calorimetry (ITC), the interaction of the guests with β-CD was examined. It was found that azo-PEG-azo formed complexes with β-CD in a 1:2 ratio, with association constant of 8.62 × 10^3^ M^−1^ (Supplementary Fig. 13). The photoisomerization of azo-PEG-azo was also investigated (Supplementary Fig. 14). We demonstrated this homo-bifunctional cross-linking agent served as ‘reversible cell glues^38^' could induce adhesion and aggregation of β-CD-modified cells. Cell assembly and disassembly could be reversibly manipulated with light irradiation (Fig. 4a). The β-CD modified MCF-7 cells were divided into two subsets. For tracking purposes, the two subsets of cells were labelled with green or red dyes respectively, so that the cell clustering could be easily observed. When mixed together at 1:1 ratio in the absence of azo-PEG-azo, cells were well separated and no aggregates were observed. However, addition of azo-PEG-azo caused apparent cell aggregation (Fig. .4b; Supplementary Fig. 15a). Both green- and red-stained cells were present in the same cell cluster. To further confirm that the cell assembly was attributed to supramolecular interactions, a series of control experiments were added. As shown in Supplementary Fig. 15, cell binding did not occur without the proper complementary host-guest pair. Moreover, competition experiments, using adamantane, were also performed. β-CD-modified cells were firstly exposed to adamantine, followed by azo-PEG-azo. As a result, no cell aggregation was observed, which indicated azobenzene binding could be blocked by adamantine. All above results confirmed that the cell binding was attributed to supramolecular interactions.

**Figure 5:**
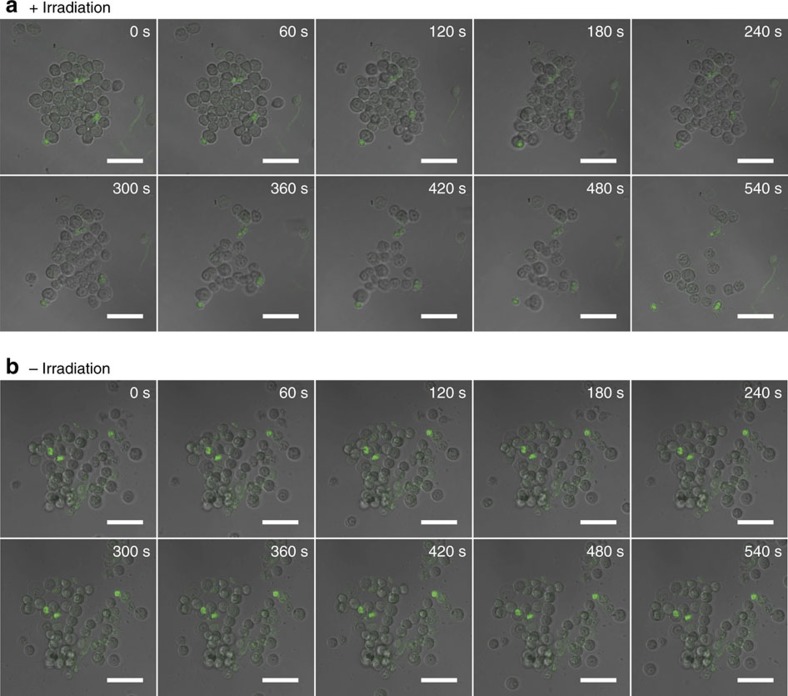
Real-time monitoring of light-induced cell disassembly. Real-time CLSM images of cell cluster (**a**) treated with or (**b**) without ultraviolet irradiation (365 nm, 15 W). Flow at 1 ml min ^−1^ was applied for the process. Scale bars, 50 μm.

Due to the remarkable difference in the binding affinity between β-CD and *trans*-azobenzene (high) and β-CD and *cis*-azobenzene (low), photoinduced dispersion and re-aggregation of the cells could be achieved by ultraviolet irradiation, followed by Vis irradiation (Fig. 4b). This novel property of light-controlled reversible assembly was attributed to the noncovalent nature of host–guest interactions. To give more details, real-time monitoring of light-induced cell disassembly was performed. The cell aggregates were irradiated with ultraviolet light (365 nm, 15 W), and the image was recorded every 60 s. As shown in Fig. 5a, looseness and disassembly of the cell cluster were gradually observed with time increasing. When time came to 540 s, the cell disassembly was completed. The control experiment without ultraviolet irradiation was also carried out at the same condition (Fig. 5b). Although the morphology of the cell cluster was changed, no obvious cell disassembly was observed. The time scanning images clearly demonstrated the process of light-induced cell disassembly. At last, quantitative analysis of the photo-controlled manipulation of cell assembly was added using flow cytometry (Fig. 4c). In the absence of azo-PEG-azo, the flow cytometry data did not show any aggregates in the upper right quadrant of the scatters, which meant most cells were separated. As expected, a new data cluster representing cellular aggregates appeared in the scatter when addition of 20 μM azo-PEG-azo. The cluster represented 47.2% of cells formed aggregates. On ultraviolet irradiation, the data cluster disappeared, with 8.9% cell aggregates remained, which meant photo-induced dispersion of the cells was achieved. We have also quantified the percentage of azo-PEG-azo bound on the same cell surface versus trans-cellular. The results showed about 71% of azo-PEG-azo used for trans-cellular binding (Supplementary Fig. 16).

### Heterotypic cell adhesion and cell-based therapy

**Figure 6:**
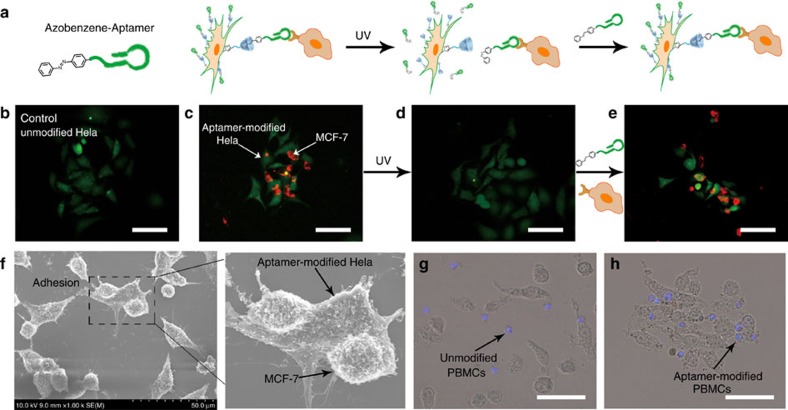
Fluorescence images and SEM images of heterotypic cell adhesion. (**a**) Azobenzene-labelled-aptamers (azo-aptamer) anchored on the cell surface could act as targeting ligands that specifically recognized their target cells and induced cell–cell adhesion. MUC 1 aptamer-modified Hela cell (green) could recognize the target cells MCF-7 (red) and form a cell-aptamer-cell assembly. (**b**,**c**) Fluorescence images of heterotypic cell adhesions between unmodified Hela (**b**), modified Hela (**c**) and MCF-7. (**d**) After ultraviolet irradiation (365 nm, 15 W, 10 min) and washing with PBS, MCF-7 cells were released from the surface of Hela cells. (**e**) Hela cells could be once again modified with azo-aptamer and used for cell-cell adhesion. (**f**) SEM images of cellular interactions. Cell–cell adhesion was clearly observed. (**g**,**h**) Microscope images of heterotypic cell adhesions between unmodified PBMCs (**g**), modified PBMCs (**h**) and MCF-7. Scale bars, 50 μm.

Furthermore, we aimed at endowing cell-cell contacts with targeting property. Considering the high binding affinity of aptamer to their targets, we prepared azo-aptamer as a photo-switchable recognition component and anchored it on the cell surface as binding agents to dynamic control over cell–cell interactions (Fig. 6a). In this experiment, aptamer MUC 1, which targets mucin 1 protein expressed on epithelial cancer cells (MCF-7; MUC 1+), was used for testing^35^. The aptamer exhibited high affinity towards their target cells by mimicking native cell-surface ligand–receptor interactions. We expected that Hela cells modified with MUC 1 aptamer would recognize their target cells MCF-7 and form a cell-aptamer-cell assembly. For easily distinguishing, Hela cells and MCF-7 cells were stained with green and red dyes respectively. As can be seen from Fig. 6b,c, after washing, MCF-7 cells could not adhere to unmodified Hela cells. By contrast, heterotypic cell adhesions were successfully observed between MCF-7 cells and MUC 1 aptamer-presenting Hela cells. Scanning electron microscopic (SEM) images gave detailed three-dimensional morphology of cellular interactions. The cellular morphologies were well maintained, and direct contact at cell–cell interfaces was clearly observed (Fig. 6f). As mentioned above, an advantage of introducing photo-responsive host-guest recognition is the potential for controlled photoirradiation to reverse the linkage. ultraviolet irradiation induces the photoisomerization of *trans*-isomer to *cis*-isomer. Since *cis*-azobenzenes cannot form inclusion complexes with β-CD, azo-aptamers are no longer displayed on the cell surface, resulting in disassociation of cell-cell contacts. As shown in Fig. 6d, on ultraviolet irradiation, MCF-7 cells were released from the surface of Hela cells. We further demonstrated that β-CD-labeled Hela cells could be once again modified with azo-aptamer and used for cell–cell adhesion (Fig. 6e). Although aptamer has also been used as a binding agent for cell–cell contacts^12^, our attractive method exhibited obvious advantage over the previous reported irreversible effects, in which the altered structure could not be reused. At last, we illustrated an actual use by applying our method toward directing PBMCs to induce target cell apoptosis. Human PBMCs were obtained through density gradient centrifugation and modified with aptamer MUC 1 using the above labelling strategy. The resulted aptamer-modified PBMCs or unmodified PBMCs were co-incubated with MCF-7 cells, respectively, and shaken at 37 °C. The cell–cell contacts were viewed using optical microscope. As can been seen from Fig. 6g,h, compared with unmodified PBMCs, aptamer-modified PBMCs could effectively adhere to the target MCF-7 cells and form T cells–cancer cells assembly. We also estimated the efficiency of PBMCs-induced cell apoptosis by lactate dehydrogenase release assay^16^, which indicated aptamer-modified PBMCs showed enhanced cytotoxicity to MCF-7 cells (Supplementary Fig. 17).

## Discussion

We engineered cell membranes with β-CD for dynamic control of cell-cell interactions using a combination of metabolic glycan labelling and bio-orthogonal click reaction. Furthermore, the aggregation and dispersion of cells were mediated by a fully reversible photo-responsive molecular recognition process. Three important features are associated with our photo-switchable cell surface engineering system: (1) this method allows modulating intercellular contacts in space and time; (2) cell–cell adhesion can be reversible controlled by light; (3) due to the presence of β-CD, we envision that a series of stimuli-responsive host-guest recognition can be introduced to meet multi-level demand. Our design opens a new avenue to control contact-dependent cell–cell reversible interactions and will promote further studies on cell communications.

## Methods

### Cell labelling experiments

N-azidoacetylgalactosamine (Ac_4_GalNAz) was purchased from Invitrogen (Eugene, OR, USA). FAM alkyne and Copper(II)-TBTA complex (10 mM in 55% aq. dimethylsulfoxide) were purchased from Lumiprobe. MCF-7 cells/Hela cells were cultured using the general DMEM medium containing 50 μM Ac_4_GalNAz for three days to enrich the azido groups in O-linked glycoproteins. The Ac_4_GalNAz-labelled cells were washed three times with 1X PBS, then incubated with PBS containing 50 μM Copper(II)-TBTA complex, 2 mM sodium ascorbate, 25 μM FAM alkyne (or 50 μM alkynyl-PEG-β-CD) in the dark at rt for 10 min, followed by three washes before being used for the following experiments. The β-CD modified cells were incubated with azo-DNA-FAM (25 μM) for 60 min, followed by three washes before fluorescence imaging and flow cytometry analysis. Fluorescence imaging experiments were performed on a Zeiss LSM700 confocal laser scanning microscope. Flow cytometry analysis was performed on BD FACS Aria. For experiment of attaching β-CD-labelled cells to an azobenzene-patterned substrate, the β-CD-labelled cells were detached from 6-well plates by incubation with 1 mM EDTA-PBS for 10 min at 37 °C. Then cells were incubated with the modified substrates for 4 h. The unbound cells were gently removed by rinsing with buffer for 1 min. Cells were stained by AM fluorescent dye for 10 min and viewed using an Olympus BX-51 optical system microscope (Tokyo, Japan). For ultraviolet triggered release, cell-attached substrate were irradiated with ultraviolet light (365 nm, 15 W) for 10 min^39^. After that, the substrate was gently washing with PBS for 15 s to remove the released cells. Then it was measured with fluorescence images to monitor the cell release.

### Cell assembly and disassembly

For investigating reversible manipulation of cell assembly and disassembly with the homobifunctional guest molecule (azo-PEG-azo), the β-CD-labelled cells were detached from 6-well plates by incubation with 1 mM EDTA-PBS for 10 min at 37 °C. Green-stained and red-stained β-CD-modified MCF-7 were mixed together at a 1:1 ratio in the presence of different concentrations of azo-PEG-azo, and shaken at 300 rpm for 60 min at 25 °C. Aliquots were analysed by optical microscope. For ultraviolet triggered disassembly, cell aggregates were irradiated with ultraviolet light (365 nm, 15 W) for 10 min and shaken in the dark at 300 rpm for 30 min at 25 °C. Aliquots were analysed by optical microscope. Flow cytometric analysis was performed on BD FACS Aria.

### Heterotypic cell adhesions

The β-CD-modified Hela cells were incubated with 20 μM azobenzene labelled MUC 1 aptamer for 20 min. After washing, MCF-7 cells were added for heterotypic cell adhesions. For easily distinguishing, Hela cells and MCF-7 cells were stained with green (fluorescein) and red (lissamine rhodamine B) dyes respectively. For ultraviolet triggered release, the sample was irradiated with ultraviolet light (365 nm, 15 W) for 10 min. After that, the substrate was gently washing with PBS for 15 s to remove the released cells.

Human peripheral blood mononuclear cells (PBMCs) were obtained through density gradient centrifugation and cultured with RPMI-1640 media, followed by modified with aptamer MUC 1 through the above labelling strategy. Briefly, PBMCs were cultured with RPMI-1640 containing 50 μM Ac_4_GalNAz for three days to enrich the azido groups. The azido-labelled cells were washed three times with PBS, then incubated with PBS containing 50 μM Copper(II)-TBTA complex, 2 mM sodium ascorbate, 50 μM alkynyl-PEG-β-CD in the dark at rt for 10 min, followed by three washes. Then the β-CD-modified PBMCs were incubated with 20 μM azobenzene labelled MUC 1 aptamer for 20 min. After washing, the resulted aptamer-modified PBMCs (or unmodified PBMCs) were added to each well of the 6-well plate containing MCF-7 cells. MCF-7 cells and PBMCs were then allowed to incubate together at 37 °C for one hour (shaken at 300 rpm) and further longer. The cell-cell contacts and resulting cell lysis were viewed using an Olympus BX-51 optical system microscope (Tokyo, Japan). Measurement of cytotoxicity of MCF-7 cells on incubation with aptamer-modified or unmodified PBMCs was done using a non-radioactive cytotoxicity assay (CytoTox 96® Non-Radioactive Cytotoxicity Assay)^16^ that measured the amount of lactate dehydrogenase (LDH) enzyme following cell lysis.

### SEM measurement

To gain more insight on the details, SEM characterization was included in our study. The specimens were fixed with 4% glutaraldehyde for 3 h. Next, the specimens were washed with sterile water for three times and then were dehydrated by addition of ethanol in a graded series (15% for 15 min, 30% for 15 min, 50% for 15 min, 70% for 15 min, 80% for 15 min, 90% for 15 min and 100% for 15 min) and then treated with tert-butanol. After drying under vacuum, the specimens were coated with platinum and examination on SEM.

### Data availability

The authors declare that the data supporting the findings of this study are available from the corresponding author on request.

## Additional information

**How to cite this article:** Shi, P. *et al*. Spatiotemporal control of cell–cell reversible interactions using molecular engineering. *Nat. Commun.* **7,** 13088 doi: 10.1038/ncomms13088 (2016).

## Supplementary Material

Supplementary Information Supplementary Figures 1-18, Supplementary Methods, Supplementary References
