# Supplementary material for: Spatiotemporal control of cell–cell reversible interactions using molecular engineering
Source: Nat Commun. 2016 Oct 6;7:13088. doi: 10.1038/ncomms13088 (PMC5059747; doi:10.1038/ncomms13088)
Supplement: Supplementary Information — Supplementary Figures 1-18, Supplementary Methods, Supplementary References [file ncomms13088-s1.pdf]

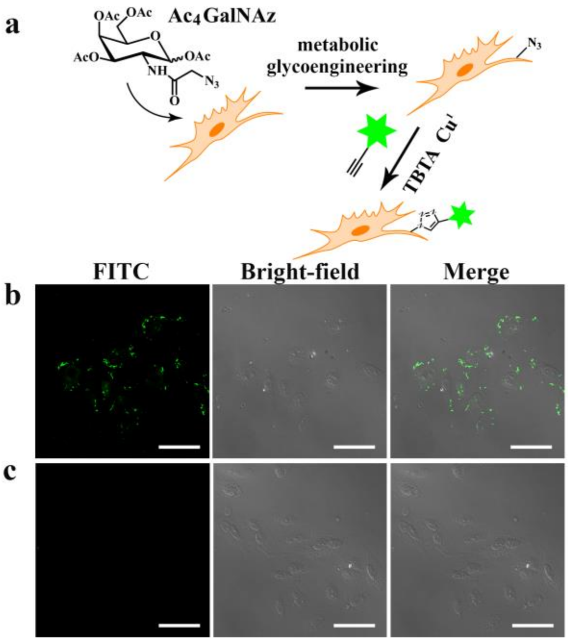

Supplementary Figure 1. Cell-surface labeling with FAM alkyne probes. (a) Outline of the labeling experiment. MCF-7 cells were metabolically labeled with Ac4GalNAz for 3 days and then reacted for 10 min with 25 μM FAM alkyne using TBTA-assisted CuAAC. (b and c) Confocal fluorescence images of MCF-7 cells treated with (b) or without (c) Ac4GalNAz, followed by FAM alkyne tagging. Scale bars, 50 μm.

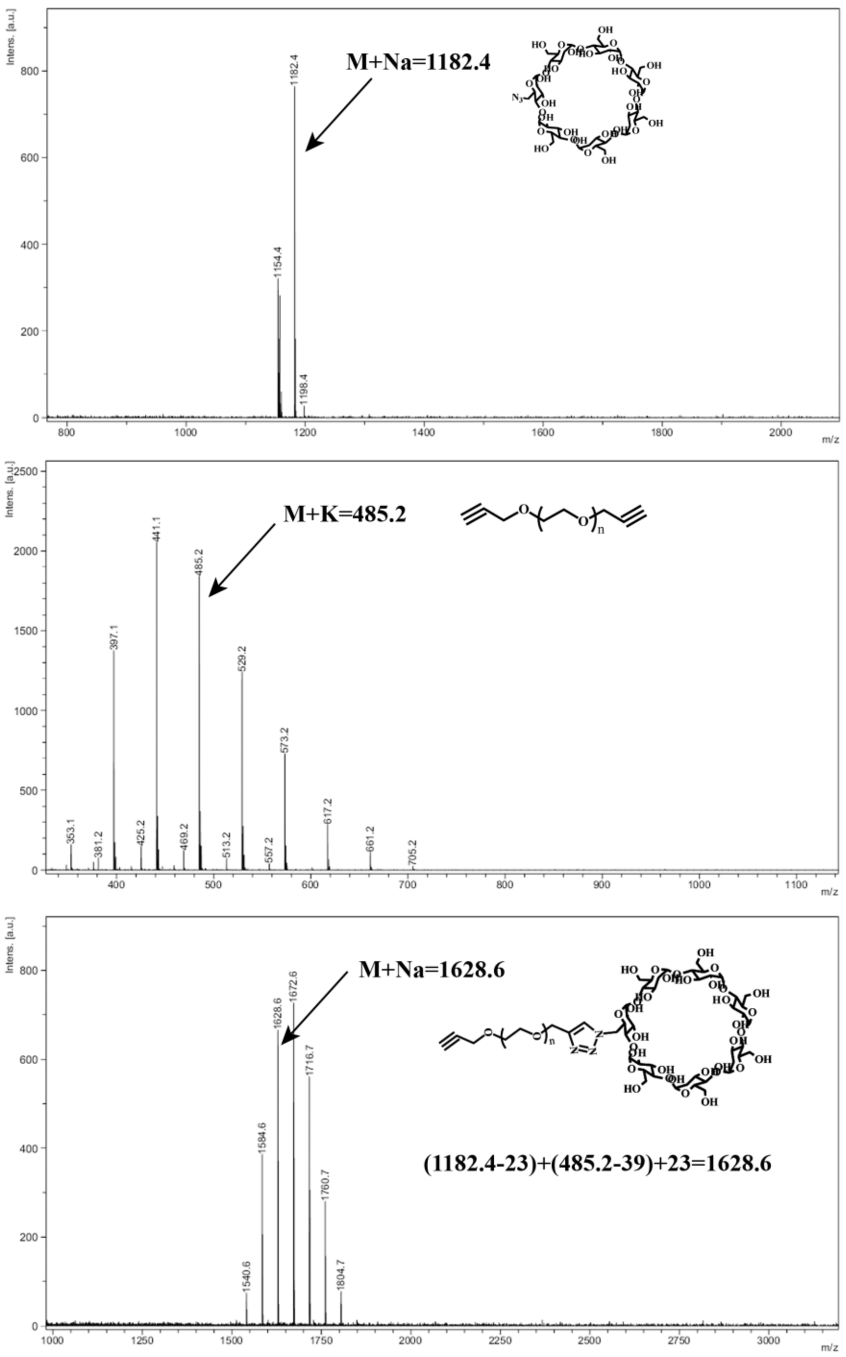

Supplementary Figure 2. MALDI-TOF MS of mono-azide β-CD, alkyne-PEG-Alkyne and alkynyl-PEG-β-CD. MALDI-TOF MS confirmed the conjugation of one molecule of β-CD to Alkyne-PEG-Alkyne. Due to the disperse PEG lengths in the compound, several peaks appear 44 mass units apart.

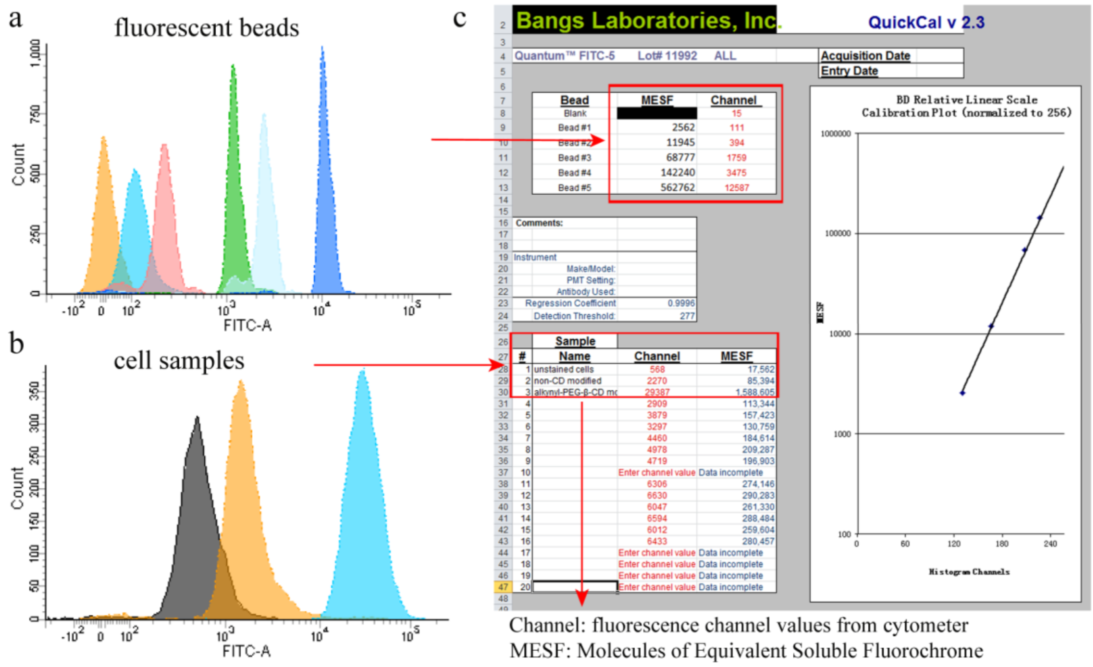

Supplementary Figure 3. Quantitative analysis using fluorescent beads and standards. a) Flow cytometry analysis of standard beads with known fluorescein molecule density. b) Flow cytometry analysis of stained cell samples at the same fluorescence settings as standard beads. c) Quantitating the fluorophore density of stained cell samples using QuickCal® v.2.3 analysis template (Bangs Laboratories, Inc.).

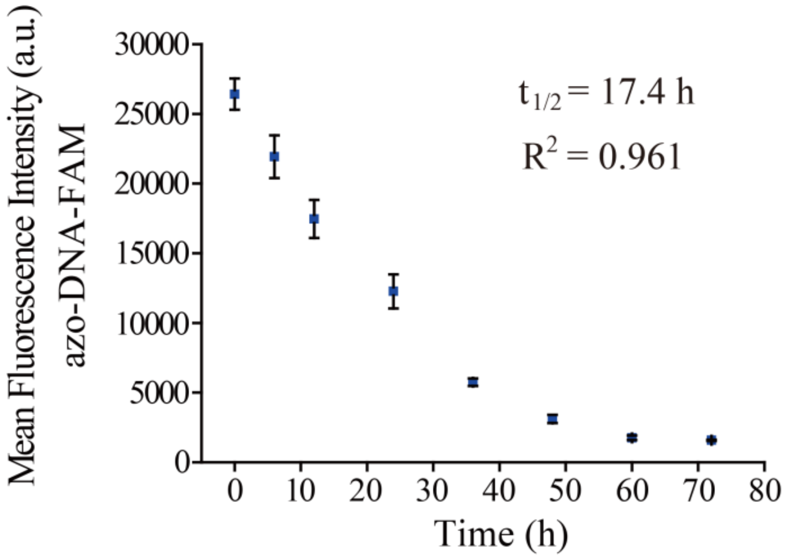

Supplementary Figure 4. Half-life of β-CD on cell surface. To determine the half-life of β-CD on cell surface, we labeled remaining surface-associated β-CD with azo-DNA-FAM over 72 h. In detail, β-CD-midified cells were incubated at 37 ºC. An aliquot of cells were removed at the indicated time and labeled with azo-DNA-FAM. Cells were washed and analyzed by flow cytometry. Experiments were performed in triplicate and half life was calculated by best fit to the equation $y = y_0e^{-kt}$. The results showed that the surface half-life was 17.4 h.

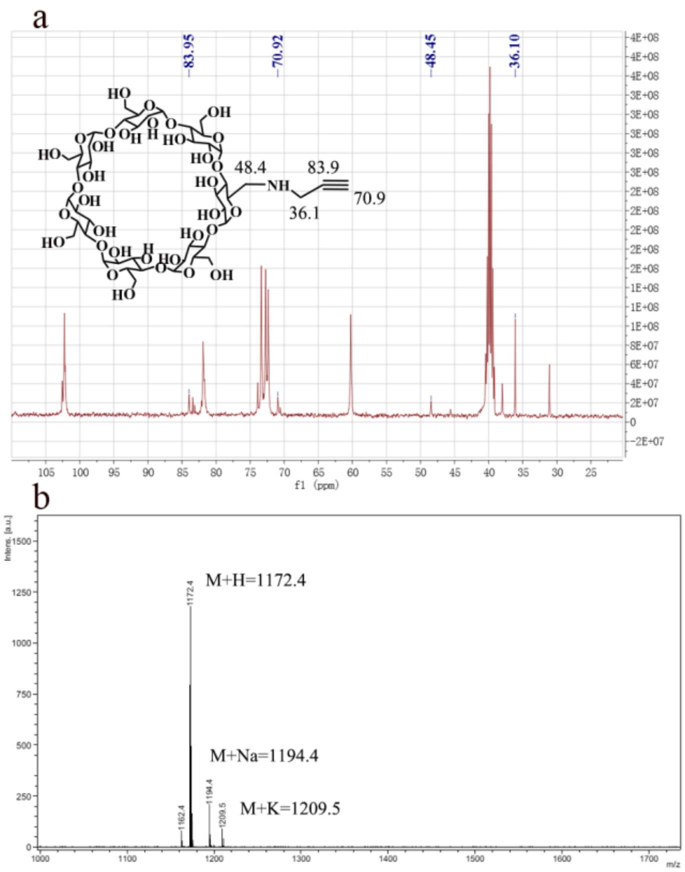

Supplementary Figure 5. ^{13}C NMR spectrum (a) and MALDI-TOF MS (b) of alkynyl-β-CD.

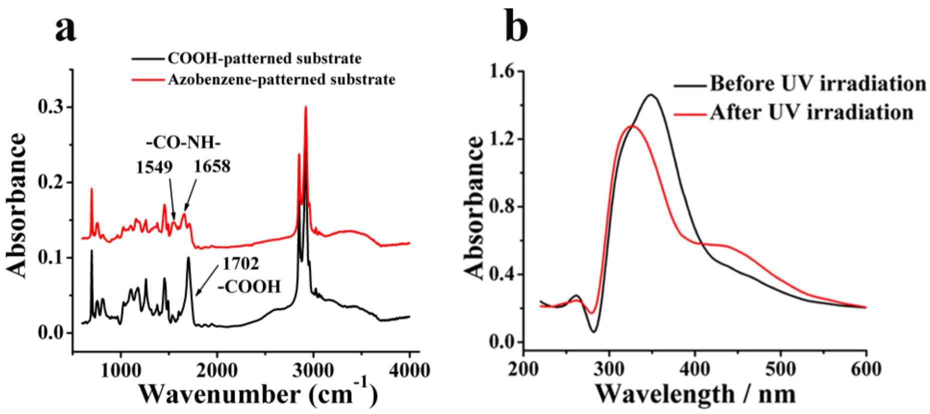

Supplementary Figure 6. ATR-FTIR spectra and UV-vis absorption spectra of azo-patterned substrate. a) ATR-FTIR spectra of carboxyl-patterned substrate and azo-patterned substrate. The –CO–NH– (1658 cm^{-1}, 1549 cm^{-1}) vibrations in the spectrum indicated that aminoazobenzene was covalently grafted onto carboxyl-patterned substrate successfully. b) UV-vis absorption spectra of azobenzene-patterned substrate before and after UV irradiation (365nm, 10min). Upon UV irradiation, the absorption at 350 nm decreased apparently. Meanwhile, the absorption at 450 nm increased.

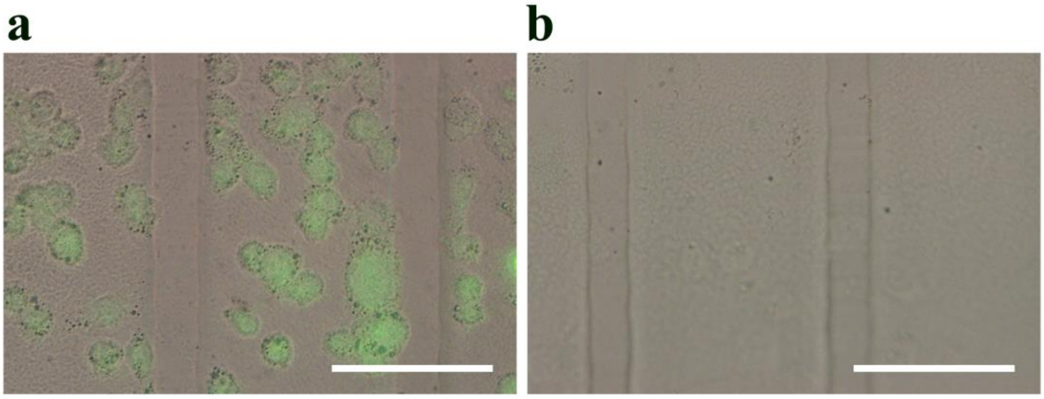

Supplementary Figure 7. Microscope images describing selective cell attachment. The β-CD-labeled cells (a) could selectively attach to trans-azobenzene-patterned regions through host–guest interactions. By contrast, unmodified cells (b) did not attach to the surface. Scale bars, 50 μm.

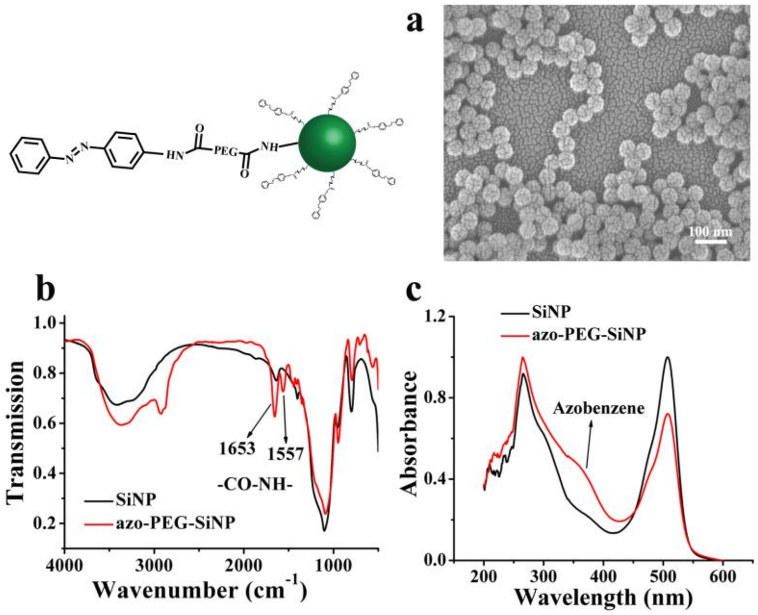

Supplementary Figure 8. Characterization of azo-PEG-SiNP: a) SEM image indicated the size of SiNP was about 50 nm. FTIR spectra (b) and UV/Vis absorption spectra (c) of SiNP and azo-PEG-SiNP. The –CO–NH– (1653 cm^{-1}, 1557 cm^{-1}) vibrations in the FTIR and the absorbance at 350 nm in the UV/Vis indicated that aminoazobenzene was covalently grafted onto SiNP successfully.

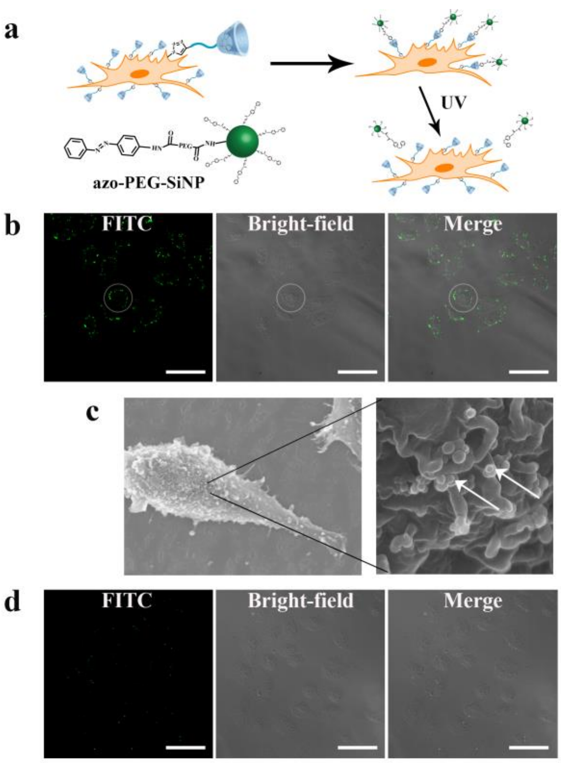

Supplementary Figure 9. Fluorescence images and SEM images describing reversible binding of azo-PEG-SiNP to the β-CD-modified cell surfaces. a) Outline of reversible binding of azo-PEG-SiNP to the β-CD-modified cell surfaces. b, d) Confocal fluorescence images of β-CD-labeled MCF-7 cells treated with azo-PEG-SiNP (b), then UV irradiation (d). Scale bars, 50 μm. c) SEM image confirmed attachment of nanoparticles on the cell surfaces.

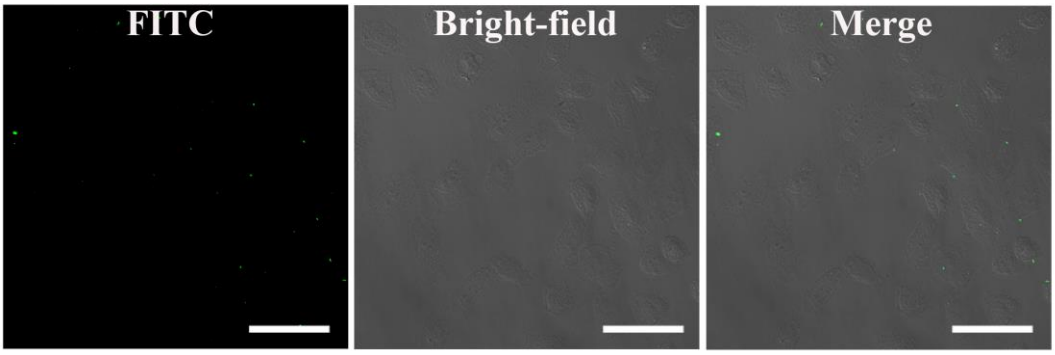

Supplementary Figure 10. Fluorescence images of non-labeled cells treated with azo-PEG-SiNP. azo-PEG-SiNP showed minimal binding to non-labeled cells. Scale bars, 50 μm.

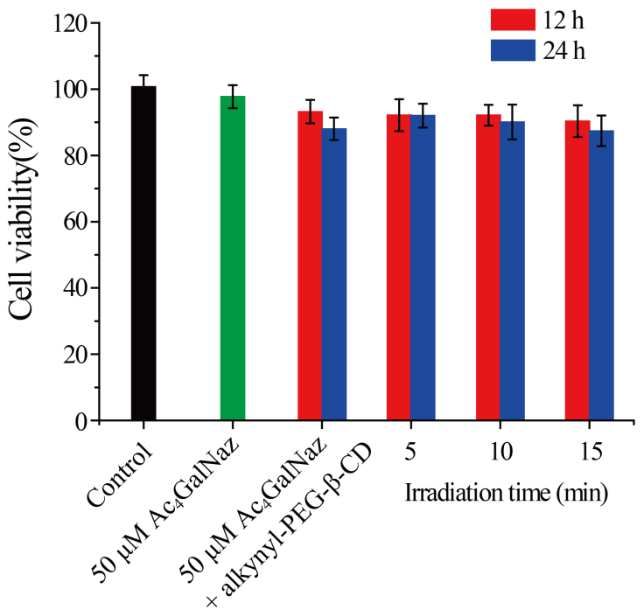

Supplementary Figure 11. Viability of MCF-7 cells after treatment of Ac_4GalNAz, alkynyl-PEG-β-CD, and UV irradiation (365 nm, 15 W) for different time. As shown in Fig. S17, azide modification had little effect on cell viability at a nontoxic concentration of 50 $\times 10^{-6}$ M. The subsequent treatment with alkynyl-PEG-β-CD resulted in slight decrease of cell viability. Besides, the viability of the cells did not change obviously when treated with 10 min UV irradiation (365 nm, 15 W). Data were presented as mean ± s.d (n=3).

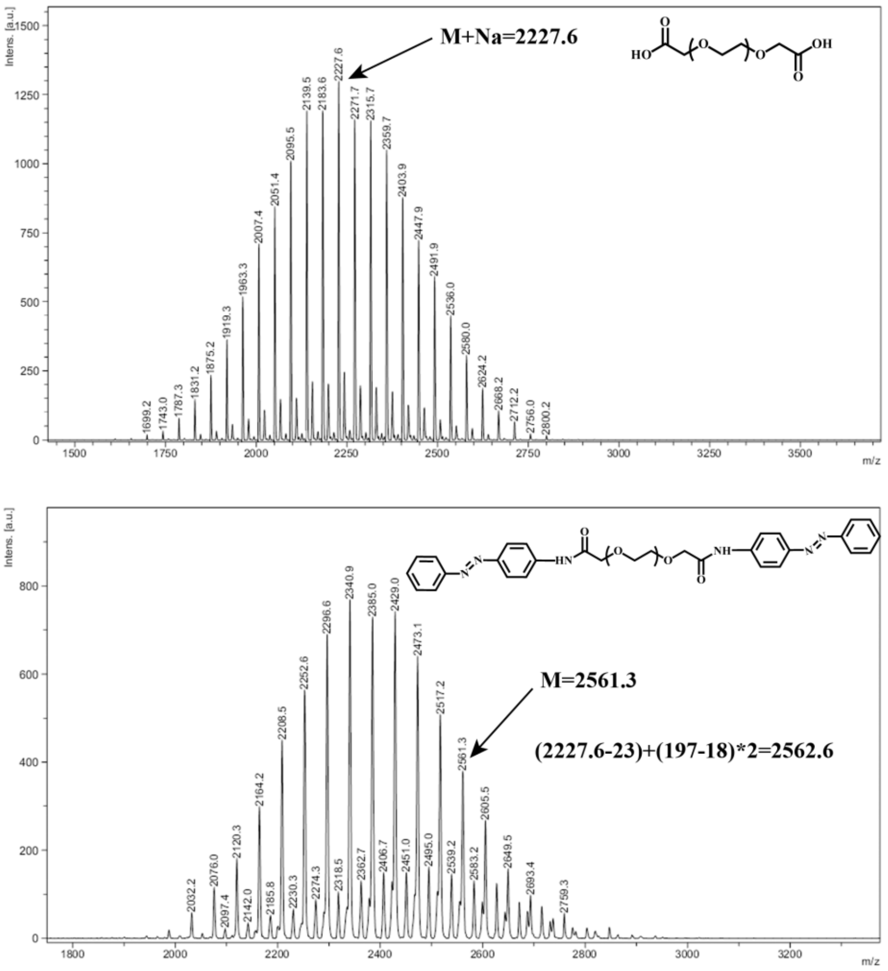

Supplementary Figure 12. MALDI-TOF MS of poly(ethylene glycol) bis(carboxymethyl) ether and azo-PEG-azo. MALDI-TOF MS confirmed the conjugation of two molecules of p-aminoazobenzene to the poly(ethylene glycol) bis(carboxymethyl) ether. Due to the disperse PEG lengths in the compound, several peaks appear 44 mass units apart.

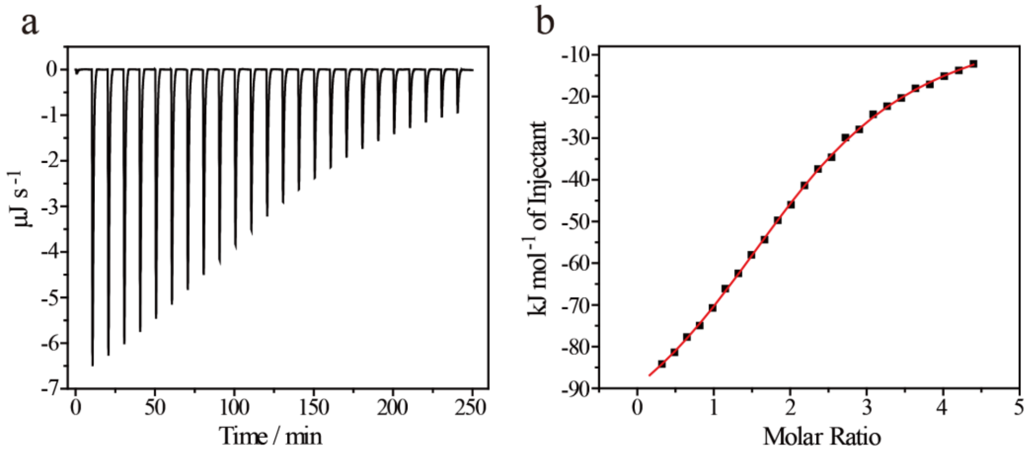

Supplementary Figure 13. ITC data corresponding to the host-guest interaction of azo-PEG-azo with β-CD. (a) Injection peaks and (b) integration of the injection peaks.

ITC assays were performed on a NANO ITC System (TA Instruments Inc., New Castle, Delaware, USA). Titrations were performed in 10 mM HEPES buffer. Injections of 10 μl of 4.5 mM azo-PEG-azo were added from a computer-controlled microsyringe at an interval of 600 s into a β-CD (0.2 mM) solution, with stirring at 400 rpm at 25 °C. The experimental data were analyzed with NanoAnalyze software (TA Instruments Inc.) and were fitted to an independent model concurrently with a blank constant model to adjust for the heat of dilution. All measurements were obtained from 25 injections of 4.5 mM azo-PEG-azo into 1400 μl of β-CD in 10 mM HEPES buffer at 25 °C. Each heat-burst curve was the result of a 10 μl injection of azo-PEG-azo into the β-CD solution.

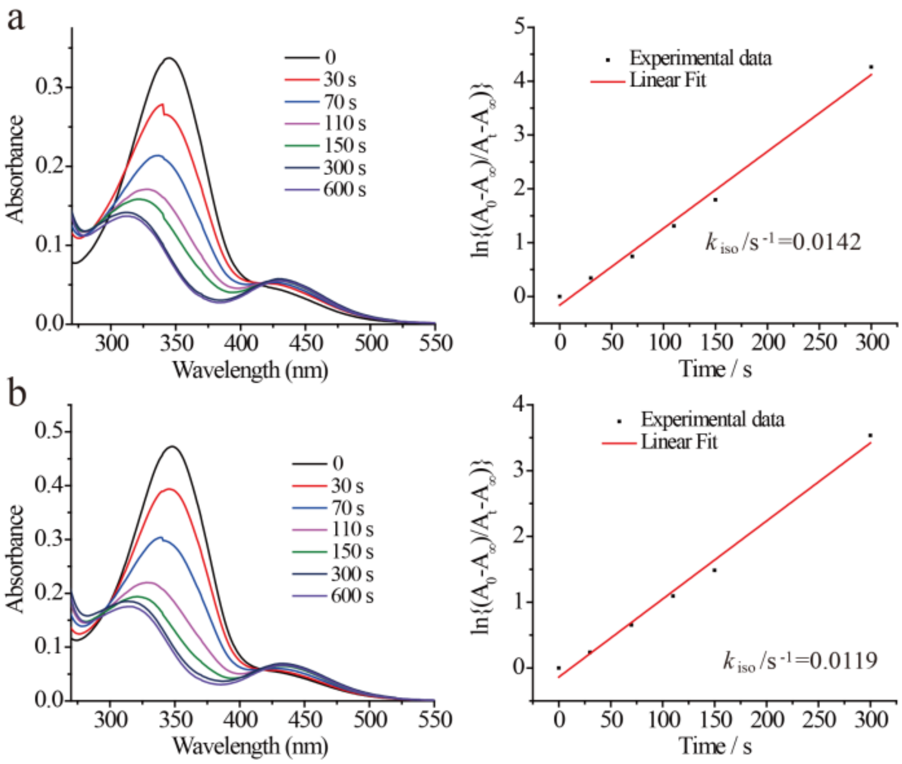

Supplementary Figure 14. Absorbance evolution of azo-PEG-azo a) in aqueous solution and b) in β-CD as a function of irradiation time. The right inset shows a linear fit of equation using the absorption data at 350 nm.

The photoisomerization of azo-PEG-azo was also investigated. We have measured absorption spectra of azo-PEG-azo in aqueous solution and in β-CD at different times of UV irradiation, and calculated the isomerization rate constants k_{iso} according to the following equation. Although the isomerization rate constants k_{iso} decreased in the presence of β-CD, azobenzene could change its configuration under the UV irradiation for 10 min.

$$\ln\frac{A_0 - A_\infty}{A_t - A_\infty} = k_{iso}t$$

k_{iso} was the rate constant in $s^{-1}$and t was time in s. A_0, A_∞, and A_t were the observed absorbances of the solution at zero time, at the end of the reaction, and at time t, respectively.

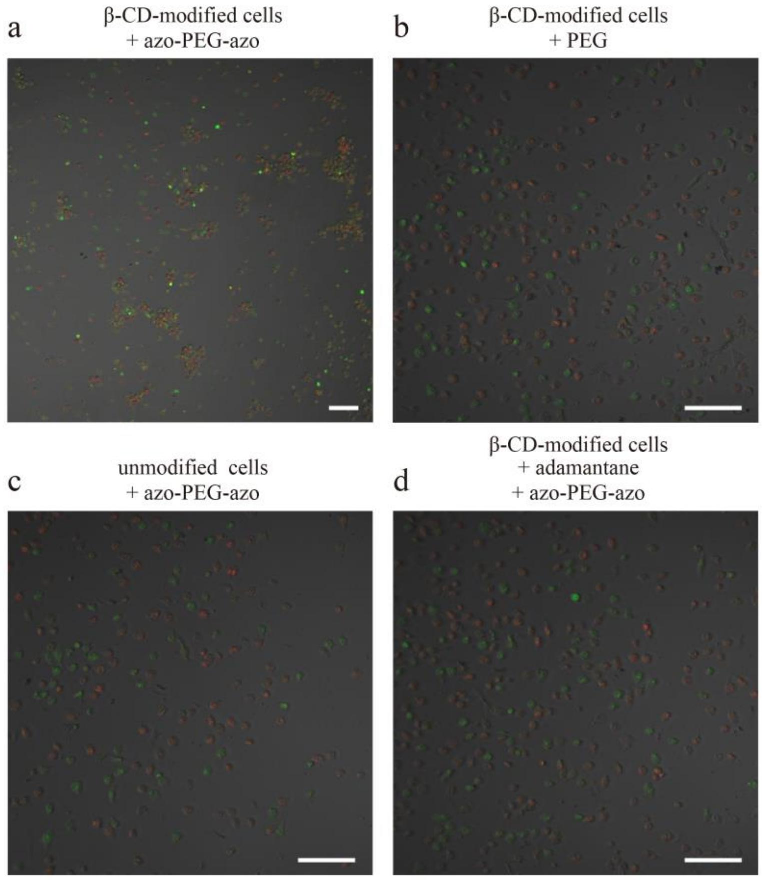

Supplementary Figure 15. CLSM images describing cell-cell contacts. (a) Addition of azo-PEG-azo caused apparent aggregation of β-CD-modified cells. (b) Treating β-CD-modified cells with PEG (Mw 2,000 Da) didn't cause any cell aggregation. (c) Unmodified cells did not form any aggregates when treating with 20 μM azo-PEG-azo. (d) β-CD-modified cells were firstly exposed to adamantine, and subsequently treated with azo-PEG-azo. As a result, no cell aggregation was observed. Scale bars, 100 μm.

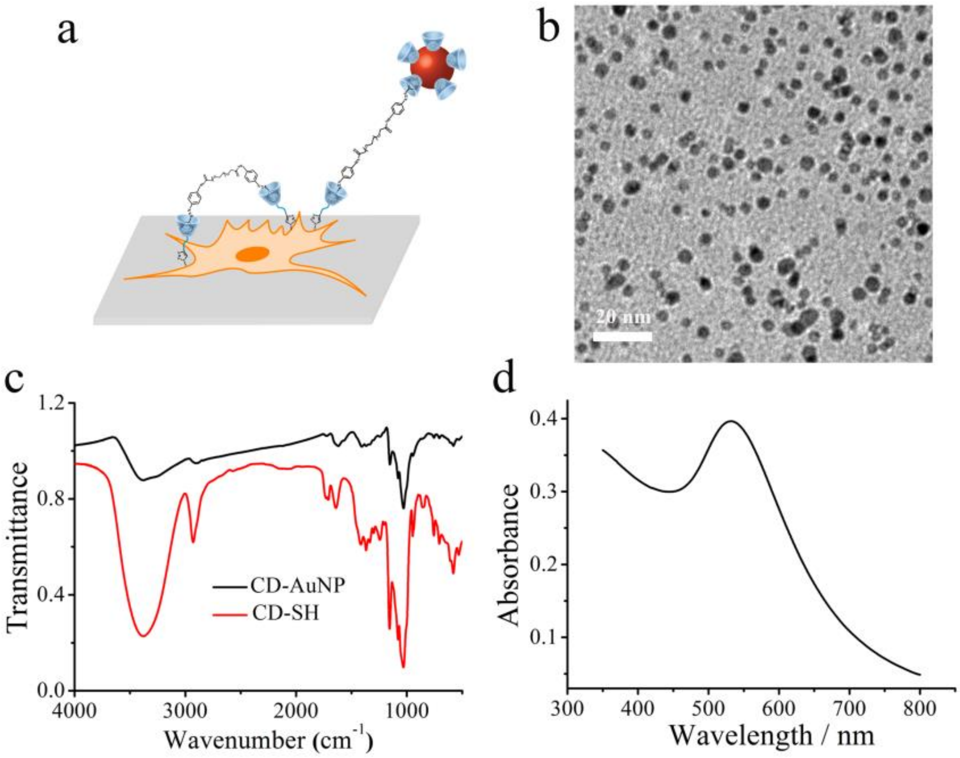

Supplementary Figure 16. (a) Schematic illustration describing quantification of the percentage of trans-cellular azo-linkers. (b)TEM image, (c) FTIR spectra and (d) UV-vis absorption spectra of CD-AuNP.

Theoretically, these two situations (azo-linkers bound on the same cell surface or trans-cellular) existed simultaneously. It was difficult to quantify the percentage of azo-linkers used for trans-cellular, as we could not obtain the data of saturated conditions (all of azo-linkers bound on the same cell surface or used for trans-cellular). Herein, we tried to construct a model to give quantitative data (as shown in Fig. S17a).

We synthesized β-CD-modified gold nanoparticle (CD-AuNP, Fig.S17b, c, d). The diameter was 3 nm and the average particle was covered by ~10 covalently attached β-CD hosts (J. Am. Chem. Soc. 2001, 123, 11148). The number density of the particles (N) could be determined from the absorbance (A_{450}) of the hydrosol, according to the following equation (Anal. Chem. 2007, 79, 4215):

$$N = \frac{A_{450} \times 10^{14}}{d^2\left[-0.295 + 1.36\, exp\left(-\left(\frac{d-96.8}{78.2}\right)^2\right)\right]}$$

β-CD-modified cells immobilized on the culture plate were firstly treat with azo-PEG-azo, and subsequently incubated with CD-AuNP. Quantification of CD-AuNP binding could be calculated from the difference of density prior (N_0) and post (N_t) incubation:

$$N_0 - N_t = \frac{(A_0 - A_t) \times 10^{14}}{d^2 \left[-0.295 + 1.36\, exp\left(-\left(\frac{d - 96.8}{78.2}\right)^2\right)\right]}$$

Where $A_0 = 0.30005$, $A_t = 0.2934$, d = 3 nm. Therefore, N_0-N_t = 2.675×10^{12},

The cell number was 2.5×10^6, we got 1.07×10^6 CD-AuNP per cell.

Assuming that CD-AuNP and azo-PEG-azo were combined in 1:1 ratio, the percentage of azo-linkers used for trans-cellular could be calculated as:

$$\frac{1.07 \times 10^6}{1.5 \times 10^6} \times 100\% = 71.3\%$$

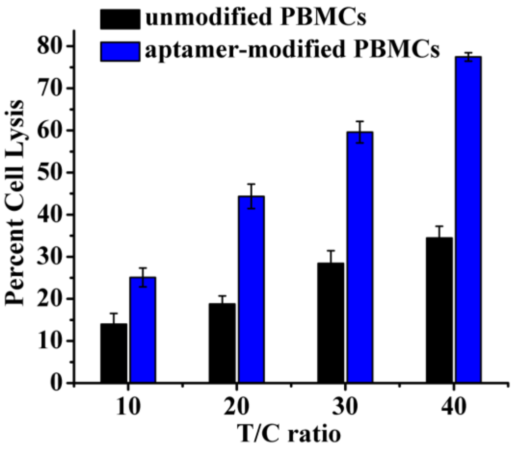

Supplementary Figure 17. Lysis of MCF-7 cells over a six-hour incubation time with aptamer-modified or unmodified PBMCs at varying T/C ratios. Data were presented as mean ± s.d (n=3).

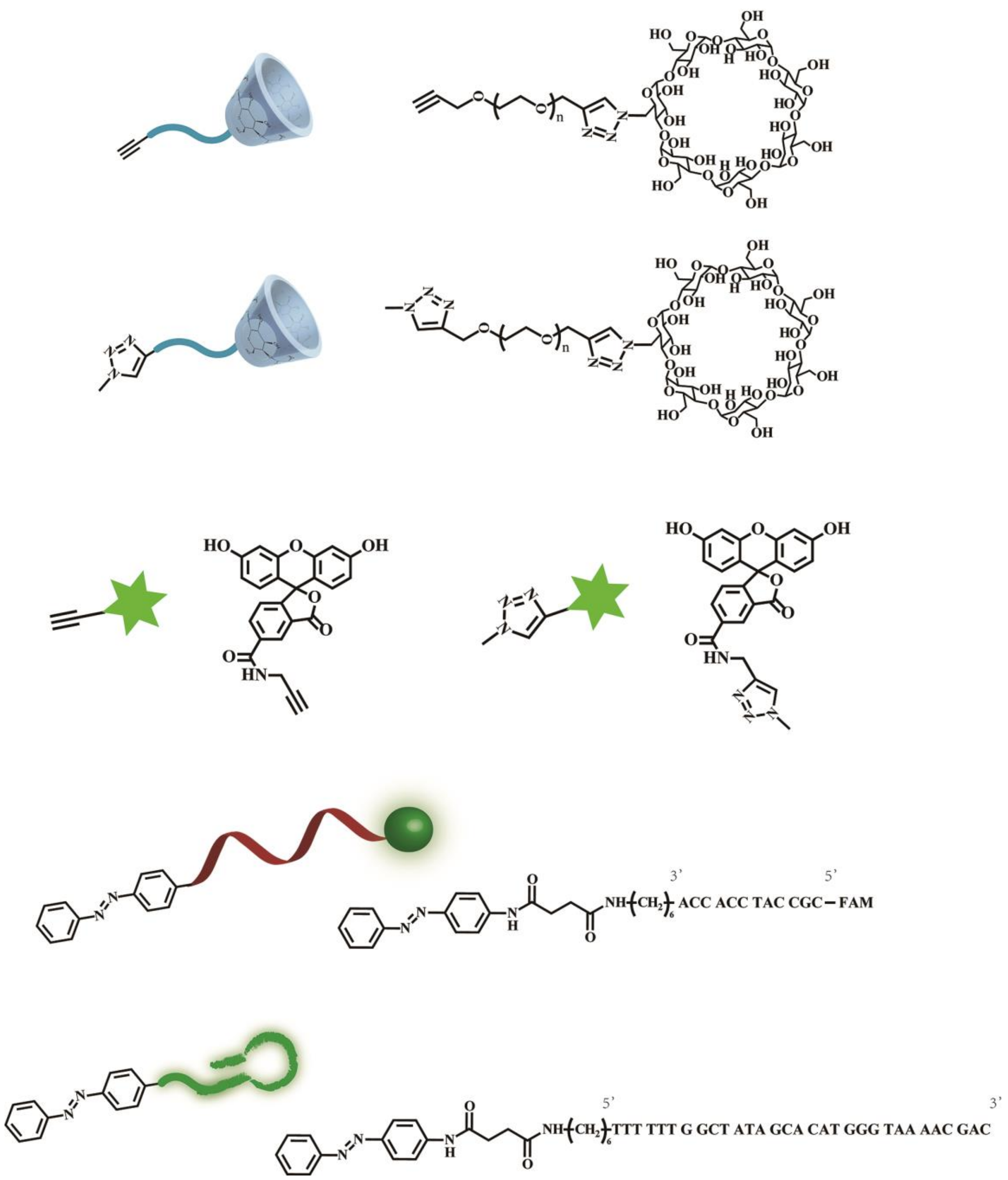

Supplementary Figure 18. Chemical structures of reagents in the scheme.

Supplementary Methods

Synthesis of alkynyl-PEG-β-CD and alkynyl-β-CD

Alkynyl-PEG-β-CD was obtained according to a previous report[1]. Mono-azide β-CD was firstly prepared through a conventional procedure[2]. Afterward, Alkyne-PEG-Alkyne (average Mw 400) (0.35 g, 0.875 mmol), Mono-azide β-CD (0.29 g, 0.25 mmol), N,N,N',N",N"-pentamethyldiethylenetriamine (52.5 µL, 0.25 mmol), and dried DMF (5 mL) were added to a Schlenk tube equipped with a magnetic stirring bar. The resultant mixture was degassed via three freeze-thaw cycles, and CuBr (35.75 mg, 0.25 mmol) was added under a nitrogen atmosphere. After stirring the mixture at room temperature for 24 h, DMF was removed under vacuum, and the product was precipitated using acetone. The obtained solid was dialyzed against water using the molecular porous memebrane (MWCO: 1000), and lyophilized for 24 h. The success of the click reaction in producing alkynyl-PEG-β-CD was confirmed by the MALDI-TOF MS.

6-Mono-TsCD (1.3 g, 1.0 mmol) and propargyl amine (2.0 mL, 29.2 mmol) were charged into a Schlenk flask (25 mL) and dissolved in 10 mL DMF. The mixture was stirred at room temperature for 48 h under the protection of N_2. The product was purified by precipitating into excess acetone for three times followed by vacuum drying. The successful preparation of alkynyl-β-CD[3] was confirmed by ^{13}C NMR spectrum and MALDI-TOF MS.

Synthesis of homobifunctional guest molecule (azo-PEG-azo)

Poly(ethylene glycol) bis(carboxymethyl) ether (average Mw 2000) (100 mg, 0.05

mmol) was reacted with EDC (69 mg, 0.36 mmol) in the presence of HOBt (60 mg, 0.396 mmol) and DMAP (10mg, 0.1 mmol) in 5 mL DMSO, stirring at room temperature for 30 min before adding to the *p*-aminoazobenzene suspension (71 mg, 0.36 mmol). The mixture was stirred at room temperature for another 24 h. The product was precipitated using diethyl ether. The obtained precipitate was dialyzed against methanol and water using the molecular porous memebrane (MWCO: 1000), and lyophilized for 24 h. The conjugation of two molecules of *p*-aminoazobenzene to the poly(ethylene glycol) bis(carboxymethyl) ether was confirmed by the MALDI-TOF MS.

Synthesis of azobenzene labeled MUC 1 aptamer

NH_2-modified MUC 1 aptamer was synthesized by Sangon Biotechnology Co. (Shanghai, China)

DNA sequences:

NH_2-C6-5’-TTT TTT G GCT ATA GCA CAT GGG TAA AAC GAC-3’

Azobenzene-COOH was firstly obtained according to a previous report[4]. Aminoazobenzene (1.97 g, 10 mmol) and succinic anhydride (1.20 g, 12 mmol) were dissolved into 25 ml distilled acetone. 0.79 g (10 mmol) anhydrous pyridine was added into the solution and the mixture was stirred for 6 h at 60 ºC. The obtained suspension was filtered and after been dried at 50 ºC for 48 h under vacuum drying, 2.85 g azobenzene-COOH (azo-COOH) was obtained.

The azobenzene-COOH was conjugated to the 5’-NH_2-moiety of the oligonucleotide, using the succinimide coupling (EDC-NHS) method[5]. Briefly, the

azobenzene-COOH was activated in the activation solution (200 mM EDC and 50 mM NHS in MES buffer) for 0.5 h. After rinsing with 10 mM phosphate buffer solution (PBS, pH = 7.8), 100 μL of 100 μM DNA was added. The mixture was stirred at room temperature for 12 h. The excessive azobenzene-COOH was subsequently dialyzed against nanopure water (18.2MΩ/cm, Millpore Co., USA) through the molecular porous memebrane tubing (MWCO: 2000, Spectrum Laboratori es, Inc. US) at 4 ºC. MALDI-TOF MS: m/z 10374.9 $[M+K]^+$. The resulting DNA solution was stored at 4 ºC for use.

Synthesis of azobenzene and PEG-modified fluorescent silica nanoparticle (azo-PEG-SiNP)

Firstly, we obtained FITC-doped silica nanoparticles: FITC was firstly linked to the coupling agent APTES by a reaction of FITC (0.5 mg) and APTES (50 μL) in 1-hexanol (2.5 mL) of under N_2 protection. Next, 0.2 mL of the resulting APTES–FITC conjugates was added to the water/oil (W/O) microemulsion solution containing cyclohexane (7.5 mL), 1-hexanol (1.6 mL), Triton X-100 (1.77 g), and deionized water (480 μL), and stirred for 15 min. TEOS (50 μL) was then added. After stirring for another 30 min, the hydrolysis of TEOS and FITC–APTES was initiated by the addition of NH_4OH (60 μL), and the mixture was stirred for 24 h. The final surface layer incorporating primary amines was formed by adding APTES (50 μL) and stirring for 24 h. Finally, the as-prepared FITC-doped silica nanoparticles were centrifuged and washed four times with ethanol and three times with deionized water.

To obtain PEG-modified fluorescent silica nanoparticle, the purified SiNP-NH_2 (30 mg) was dispersed in 10 mL DMSO. 50 mg poly(ethylene glycol) bis(carboxymethyl) ether was reacted with 20 mg NHS and 30 mg EDC in 4 mL DMSO, stirring at room temperature for 30 min before adding to the SiNP-NH_2 suspension. The mixture was stirred at room temperature for another 24 h, followed by filtration and washing with DMSO, ethanol and water, resulting in COOH-PEG-SiNP. Next, COOH-PEG-SiNP was dispersed in 10 mL DMSO, reacted with 20 mg NHS and 30 mg EDC for 30 min, followed by addition of 30 mg p-aminoazobenzene. The mixture was stirred at room temperature for another 24 h, followed by filtration and washing with DMSO, resulting in azo-PEG-SiNP.

Preparation of azobenzene-patterned substrate

Firstly, COOH-patterned substrate was obtained according to the previous report[6]. Methacrylic acid was introduced to afford carboxyl. Briefly, the COOH-patterned substrate was based on styrene-b-(ethylene-co-butylene)-b-styrene elastomer (SEBS), with poly(2-acryl-amido-2-methylpropane sulfonic acid) (PAMPS) and poly(methacrylic acid) (PMAA) brushes. A versatile two-step surface-initiated photo-polymerization (SIPP) technique was used for the preparation of COOH-patterned substrate.

For the SIPP with AMPS, flat SEBS films were immersed in an ethanol solution of benzophenone (1.5 wt %) for 30 min and then dried in vacuo under dark condition for 1 h at 25 °C. Monomer solution was prepared prior to the experiments by mixing AMPS monomer (3.2 g) in degassed DI water (36.8 mL) and then argon was bubbled

for 30 min to eliminate any oxygen. The BP-preadsorbed SEBS films were put on the slide glasses and coated with 8 wt % aqueous solution of AMPS, followed by covering with another quartz plate (0.8 mm thick). Then the sandwiched system was exposed to UV illumination (high-pressure mercury lamp, 400 W, main wavelength 380 nm) at a distance of 15 cm for 8 min at ambient temperature (22 °C). After SIPP, the samples were vigorously rinsed with deionized water and ethanol for 24 h to remove unreacted monomers and unreacted initiators. The samples were then dried in a vacuum oven for 24 h at room temperature. The so-obtained poly(AMPS)-grafted film was used for second SIPP. The second SIPP was carried out in the same manners as that mentioned above and copper grids with different shapes and sizes served as photomasks when SIPP was conducted to obtain the second patterned PMAA brushes. The obtained binary polymer brushes were referred to as COOH-patterned substrate.

Next, COOH-patterned substrate were immersed in the activation solution (200 mM EDC and 50 mM NHS in MES buffer) for 0.5 h. After rinsing with 10 mM phosphate buffer solution (PBS, pH = 7.8), aminoazobenzene was added. The mixture was stirred at room temperature for 12 h. The samples were vigorously rinsed with deionized water and ethanol for 24 h to remove the excessive azobenzene. The successful modification of azobenzene was confirmed by ATR-FTIR spectra and UV/Vis absorption spectra.

Supplementary References

1. Y. Zhang, L. Ren, Q. Tu, X. Wang, R. Liu, L. Li, J.-C. Wang, W. Liu, J. Xu and J. Wang, *Analytical Chemistry* 2011, **83**, 9651-9659.
2. J. Stadermann, H. Komber, M. Erber, F. Däbritz, H. Ritter and B. Voit, *Macromolecules* 2011, **44**, 3250-3259.
3. Q. Ma, X. Yuan, Y. Zhao and L. Ren, *RSC Adv.*, 2015, **5**, 47998-48004.
4. Y.-H. Gong, J. Yang, F.-Y. Cao, J. Zhang, H. Cheng, R.-X. Zhuo and X.-Z. Zhang, *Journal of Materials Chemistry B* 2013, **1**, 2013-2017.
5. L. Feng, W. Li, J. Ren and X. Qu, *Nano Res.* 2015, **8**, 887.
6. J. Hou, Q. Shi, W. Ye, Q. Fan, H. Shi, S.-C. Wong, X. Xu and J. Yin, *Chem. Comm* 2015, **51**, 4200-4203.
